# Gas Discharge Visualization: An Imaging and Modeling Tool for Medical Biometrics

**DOI:** 10.1155/2011/196460

**Published:** 2011-05-19

**Authors:** Nataliya Kostyuk, Phyadragren Cole, Natarajan Meghanathan, Raphael D. Isokpehi, Hari H. P. Cohly

**Affiliations:** ^1^Center for Bioinformatics and Computational Biology, Jackson State University, Jackson, MS 39217, USA; ^2^Department of Communicative Disorders, Jackson State University, Jackson, MS 39217, USA; ^3^Department of Computer Science, Jackson State University, Jackson, MS 39217, USA; ^4^Department of Biology, Jackson State University, Jackson, MS 39217, USA

## Abstract

The need for automated identification of a disease makes the issue of medical biometrics very current in our society. Not all biometric tools available provide real-time feedback. We introduce gas discharge visualization (GDV) technique as one of the biometric tools that have the potential to identify deviations from the normal functional state at early stages and in real time. GDV is a nonintrusive technique to capture the physiological and psychoemotional status of a person and the functional status of different organs and organ systems through the electrophotonic emissions of fingertips placed on the surface of an impulse analyzer. This paper first introduces biometrics and its different types and then specifically focuses on medical biometrics and the potential applications of GDV in medical biometrics. We also present our previous experience with GDV in the research regarding autism and the potential use of GDV in combination with computer science for the potential development of biological pattern/biomarker for different kinds of health abnormalities including cancer and mental diseases.

## 1. Introduction

Biometrics is the field of science which brings together biology, physiology, psychology, computer science, mathematics, statistics, and engineering. The global interest in biometrics is motivated by its accuracy, reliability, and instantaneous real-time readout. Nowadays, biometrics is penetrating into many areas of social life. For example, in education, at the K-12 level, students are being introduced to the basic notions of biometric science to wake up the interest in the next generation of researchers and scientists to this fast developing field. 

According to the classic definition, biometrics is an automated process of recognizing the individual features based on one or more specific anatomy, physiology, and psychological characteristics with the purpose of recognition, identification, and verification. Recognition is the knowledge of a previously enrolled pattern; identification is the process of determining the identity of an individual according to the pattern, whereas verification is a process by which the system confirms the existing pattern. The biometric models existing nowadays are based on fingerprint, face, iris, voice, signature, hand geometry, palm, and vascular pattern recognition. There exist other biometric models that are based on speaker recognition, dynamic signature measures, key stroke dynamics, retina recognition, gait/body recognition, and facial thermography. The main areas of biometric applications can be classified into the following four groups:

1. security biometrics to reduce frauds and control the access to restricted areas,
2. forensic biometrics, which refers to the use of biometrics for criminal and body identification,
3. convenience biometrics, which is related to maintaining the convenience level during the use of a computer or network,
4. medical biometrics, which is related to the use of biometrics in medical applications such as medical diagnosis and is based on the extraction of biomedical pattern and its association to possible diseases. Medical biometrics is emerging as a very promising and reliable method for automated medical diagnosis. Medical biometric systems have been developed to use personal features in different formats such as images, signals, and other sources in order to solve problems and provide high-performance service in the medical field.

Medical biometrics based on the gas discharge visualization (GDV) technique has been used in medicine to monitor the patients and compare their natural electro-photonic emission before and after surgeries, cancer treatments, energy healing, physiotherapy, SOQI therapy, and so on. Previous findings using GDV have demonstrated the potential capability of the GDV technique to identify the deviation from the normal functional status during the early stages of disease development as well as to monitor a transition from a disease state to normal functional state. 

The rest of the paper is organized as follows. In Section 2, we describe different types of biometrics and the areas of application, and introduce medical biometrics in Section 3. In Section 4, we discuss the possible applications of GDV in medical biometrics and our pilot studies on the applications of GDV in medical biometrics, Section 5 concludes the paper.

## 2. Types of Biometrics and Areas of Applications

The biometric signatures for the identification purposes are based on physiological and/or behavioral trait and help in the verification process. It is important to understand that all biometrics are based on probability measures. Biometrics is a mathematical model of a physical characteristic and as in all mathematical models, there is always a probability of an error. However, biometrics is the most reliable way of verification and authentication. Physiological traits used in biometrics include iris, fingerprint (including nail), hand (including knuckle, palm, and vascular), face, voice, retina, DNA, lips, earlobe, sweat pore, and even odor. Behavioral traits are based on signature, keystroke, voice, and gait [1].

Over 140 years of fingerprint comparison worldwide, no fingerprints were found to be alike not even those of identical twins. Fingerprints do not change throughout the life span, and therefore, fingerprint identification involves comparing the pattern of ridges and furrows on the fingertip as well as the minutiae points, which are ridge characteristics that happen when a ridge splits into two, or ends of a specimen print with a database of prints on file. Hand geometry authentication is often used in industrial environment. It does not require clean conditions, forms a very small dataset, and is not regarded as an intrusive kind of a test [2]. Interestingly, the authentication of the identity seems to be not descriptive enough using hand geometry. One can attain robust verification only by combining various individual features.

Iris and retina scans provide unique biometric data as it is impossible to duplicate or replicate them [3]. The pattern of blood vessels at the back of the eye and iris remains the same through lifetime. Despite being highly reliable, the disadvantage of retina scan is that the accuracy of measurement can be affected by disease such as cataracts or severe astigmatism and the equipment is not very user friendly as the subject has to be close to the camera optics leaving alone the cost of the equipment which is very high.

Face recognition is one of the most flexible identification methods as individuals are unaware that they are being scanned. Face recognition system relies on the features common to everyone's face: the distance between the eyes, width of the nose, position of cheekbones, jaw line, chin, and so on. These numerical quantities are then combined in a single code that uniquely identifies each person [4]. Some facial recognition algorithms identify faces by extracting landmarks or features from an image of a subject's face. It may analyze the relative position, size, or shape of the eyes, nose, cheekbones, and jaw and use these features further to search for other images with matching features. Other algorithms normalize a set of face images and then compress the face data in the image that is useful for face detection. A probe image is then compared with a database of face images. Three-dimensional face recognition is a relatively new trend in biometrics, and it claims to achieve an unbelievable accuracy in face recognition [5]. This technique uses 3-D sensors to capture the information about the shape of a face. The advantage of 3-D facial recognition is that it cannot be affected by changes in lighting like other techniques. It can also identify the face from a range of viewing angles, including a profile view. However, this technique could be sensitive to face expressions. 

As compared to other biometric methods such as iris or fingerprint scans, the voice recognition allows vocal information of new pin numbers or acknowledgement of license agreements to be delivered as a part of voice biometric application [6]. DNA identification is used to prove innocence or guilt, paternity testing, and the identification of missing or dead people. The coding genes constitute only 5% of the human genome; however, the repeat of identical DNA sequence can be found anywhere in noncoding sequences from one to 30 times in a row and are called variable number tandem repeats (VNTRs) [7]. The number of repeats is specific and varies from one person to another. For any given VNTR place in an individual's DNA, there will be a certain number of repeats. DNA profiling includes the isolation of the DNA from the sample, cutting the DNA up into fragments with VNTR areas, sorting them by size, and comparing the DNA fragments in different samples. The weaknesses of DNA biometrics are intrusiveness (a physical sample must be taken) and matching not occuring in real time.

A signature is another example of biometric data which is easy to gather and is not physically intrusive. Although an individual can purposely change his signature to some extent, it is still considered as basic means of identification [8]. Keystroke-biometric method is considered to be unique behavioral biometrics. It is based on dynamics of typing and depends on how an individual types his password or name [9]. Gait biometrics identifies the person by the way he/she walks, runs, or does any other motion by feet [10]. Gait biometrics would be extremely useful to identify shoplifters who pretend to look like pregnant women. The experts say that the natural way a pregnant woman walks is different from simulation, and therefore, this biometric method would significantly decrease thefts if installed in retail stores. The above-described types of biometrics have vast areas of application related to security, forensic, and convenience purposes.

## 3. Medical Biometrics

The first International Conference on Medical Biometrics held at Hong Kong, in 2008, postulated that medical biometrics is a fast developing, very promising, and reliable method for automated medical diagnosis [11]. It combines multidisciplinary technologies in biology, medicine, electronics, computing, and statistics and is different from biometrics which is a statistical approach in clinical practice. Medical biometric systems have been developed to use personal features in different formats such as images, signals, and other sources in order to address health issues and provide high-performance service in the medical field. These features are applied by combining statistical, mathematical, and engineering methods. 

Medical biometrics investigates the biological or behavioral patterns displayed by living organisms and its significant correspondence to the organism's behavior and/or health. Though medical biometrics is a relatively recent field of science, many studies have been done trying to identify unique biologic patterns pertaining to the disease profile. Usually, it involves the comparison of the sample/individual to a healthy biological pattern established as a standard, and the deviation from the standard serves as the indicator of irregularity or problem. For example, in embryology, the virtual reality biometric technique can be used to diagnose the growth and/or developmental delay of a fetus at early stages. First, the researchers measure the parameters of the embryo still present in the womb and then develop an algorithm for healthy, normal embryo by measuring width of embryo's shoulder, elbow, hip, and knee. Therefore, a virtual embryoscopy could be a biometric tool to identify the biological pattern of embryo's deviations from normal development [12]. In another study, researchers aimed to find gender differences in the amount of gingival display during smiling using two intraoral dental biometric measurements. Researchers compared the width and length of the maxillary right central incisor and the horizontal vertical overlap of the anterior teeth to determine the relationship of these two intraoral dental biometric measurements with the amount of gingival display during smiling. The study included 61 men and 66 women ranging between the ages of 23 and 52. The participants were judged on the basis of the visibility of the gingival tissues during smiling. The results of this study showed that a relatively small percentage of the subjects displayed gingival tissue when smiling. More women displayed gums than men in a 2: 1 ratio. Subjects with gingival display had significantly more horizontal and vertical overlap of anterior teeth compared to those who did not display gingivae when smiling [13]. The described study does use the dental biometrics measures, but not in the context of disease identification or dental problems of a patient. 

The literature review of genetic prognostic signatures found serious problems in the design and analysis of many studies. It has been pinpointed that research should be focused on the development of well-validated clinically useful genetic prognostic signatures that would improve therapeutic decision making beyond current practice standards. Also, the evaluation of prognostic signatures studies requires more attention [14]. Medical biometrics as a method for an automatic identification and analysis of a disease is a promising and developing field. However, sometimes the examples of medical biometrics in the medical literature refer to the problems of patient identification in the hospitals or patient identity theft or using biometric data for the identification of gender which is not relevant in the process of disease identification. The field of medical biometrics is being advanced by the research on the topics of oral cancer screening using laser-induced fluorescence, multi resolution-optical flow to correct respiratory motion in 3D PET/CT images, ultrasound imaging for bone quality assessment, texture feature extraction and classification for iris diagnosis, hepatitis diagnosis using facial color image, interactive 4D-computed tomography, recognition pattern for lung cancer, recognition, measurement, and classification of intracranial hematomas, computerized traditional Chinese medicine, and so on [11]. 

The most recent study conducted in Singapore has shown that the computerized diagnosis of energy resources of human body based on the Chinese meridian method is helpful in monitoring improvement after treatment with far-infrared irradiation, electric ionization and enzyme supplement. The system calculates the energy resource of a patient based on the electrical activity of acupuncture points and then matches the collected energy pattern to the already established norm standard, thus, showing if the energy field of a patient improved or not after treatment. Another recently developed biometric device is based on gas discharge visualization (GDV). This technique is discussed in the next section.

## 4. Gas Discharge Visualization (GDV) and Its Applications in Medical Biometrics

Gas discharge visualization (GDV) is based on electrical activity of human organism [15]. In disease condition, the electrical activity of human body is changed as compared to electrical activity in healthy state. The electron communication is altered, and therefore, the natural electrophotonic emission of the organism is changed. The GDV technique is a method that combines eastern medicine with western approach. Capturing the natural electrophotonic emission of human body, referred to as GDV-grams, allows one to identify the functional state of an individual in real time. 

The biometric method based on GDV is extracting the stimulated electrons and photons from the surface of the skin under the influence of pulsed electromagnetic field. This process is quite well studied with physical electronic methods and is known as “photoelectron emission.” The particles emitted and accelerated in the electromagnetic field emerge as electronic avalanches on the surface of the glass electrode causing the so-called “sliding gas discharge.” The discharge causes glow due to the excitement of molecules in the surrounding hydrogen, and this glow is what is being measured by the biometric method based on GDV. Therefore, short voltage pulses stimulate the electrophotonic emission concomitantly intensifying this emission in the gas discharge due to the electric field created.

The data obtained in the process of measuring of extremely weak “biophoton field” is the scientific information which may reveal the role of some electro-photon processes underlying the functional state of the body. 

In the biometric GDV method, the stimulation of electrons and photons is intensified thousand times and thus enables measurements under normal circumstances, with normal lighting, without special preparation of the objects. The design of the biometric GDV device is completely safe as the electric current that flows through is a pulse current in microamps which is not causing any depolarization of tissue or other physiological changes. Other methods using voltage pulses which last more than a few milliseconds avoid the depolarization by applying different pastes or gels. 

The process of extraction of electrons and photons in GDV method consists of two phases of capturing the images: without filter and with filter. In the initial stage, the electrons located in the outer layers of the cutaneous covering and the surrounding tissue are extracted. In the second phase, electrons from the deepest tissues in the body are included in the current flow. These electrons may have several sources.

Some of these belong to the molecular albuminous systems, and in accordance with the laws of quantum mechanics, these electrons are dispersed among all the molecules. It is as if they are “collectivized” among groups of molecules, so in principle it is impossible to say where an electron is at a given time. They form the so-called “electron cloud”, occupying a specific area in space. 

Several studies tried to determine what exactly forms the fluorescent glow (also called GDV-grams) around fingertips. Krizhanovsky et al. [16] determined that the human central nervous system plays a crucial role in the formation of skin glow in a high-intensity electromagnetic field. The ATP (adenosine Tri-phosphate) molecule acts as a neurotransmitter in the autonomous neuromuscular junctions, the ganglia, and the central nervous system. Therefore, in case of normal operation of the organism, the ATP diffusion exchange (and the electron stream) must be regular, thus ensuring the regularity and uniformity of the fluorescence (glow) that occurs during the interaction of the skin (i.e., of a finger) with the high-intensity electromagnetic field. Another study conducted by Williams [17] claims that specific structural-protein complexes within the mass of the skin provide channels of heightened electron conductivity, measurable at acupuncture points on the skin surface. Stimulated impulse emissions from the skin are also developed mainly by the transport of delocalized electrons.

In cases of imbalances and dysfunctions, immunodeficiency, or an abnormality of the microcapillary blood circulation, the transfer of electrons to the tissue is altered and inhibited, and therefore, the electron flow is not full and the stimulated current is either very small or is very irregular in time. 

Therefore, the gaps in electrophotonic emission are the indicators of the impeded transfer of electron density to the body's tissues and an abnormality in the energy supply of organs and systems.

The central nervous system (CNS) and the autonomous nervous system (ANS) regulate the activity of all the organs and systems. The loss of synchronicity and fail in autonomous regulation caused the abnormality in working coherence of organs and systems and is manifested by such symptoms as a bad state of health, disturbed sleep and digestion, abnormal perspiration, and so on. Later on, these abnormalities lead to the dysfunctions of organs; however, the degree of abnormality largely depends on the type of genetic predisposition. The ANS reacts to the commands coming from CNS and the surrounding environment and sends control signals to the organs and systems. These signals are processed at both the physiological and the endocrine and immune systems. Information is transferred to the controlling organs thus forming a Biological Reverse System and concomitantly a closed circuit. Therefore, if any of these abnormalities are taking place in one of these links, the circuit fails and desynchronization is taking place at all the most vital levels, and ANS is the first instance to reflect all the potential problems that apparently appear first in the human body.

All of the external and internal stimuli are processed by the sympathetic and parasympathetic nervous system and are reflected on the parameters of the cutaneous covering. The electrical resistance of the skin changes, both as a whole and at electropuncture points, the capillaries narrow and widen, and there is an emission of organic molecules through the pores; the nature of the transfer of electrons to the connective tissues also changes. All of these processes influence the emission of electrons from the skin and the development of electron avalanches, which is reflected in the parameters of the electrophotonic capture in the biometric method based on GDV.

The objective of GDV is to identify the functional psychoemotional and physiological state of a person using fingertips [18]. The analysis of natural electrophotonic emission is based on intensity, fractality, and area of the captured images. In GDV, the relation of the captured image to organs/organ systems is determined by the acupuncture approach, and therefore, the image is automatically divided into sectors having start angle and end angle as reference points which have been defined after Korotkov [19]. Also, GDV provides the integral parameters of entropy and autonomic tone, which are important components in the analysis of human functional state [19]. Entropy is a measure of chaos/disorder, and an increase in entropy has been postulated on the First International Congress of Systemic Medicine as a manifestation of sickness, negative impact of chemical, biological, physical, or emotional stress, and chronic degenerative disease [20]. The Congress also mentioned that the treatment of sickness should consider the reduction of entropy in the system. In GDV software, entropy is calculated based on the comparison of the captured image with the calibration values of the standard image of a specially designed cylinder which consists of the mix of titanium with other stable metal. Therefore, the emission of the calibration object in electromagnetic field is stable and homogenous. Autonomic tone is calculated as a difference between the assigned values of the sympathetic nervous system and parasympathetic nervous system and is a good indicator of a stress level [21]. Autonomic tone may serve as an indirect indicator of cognitive activity as well. 

**Figure 1:**
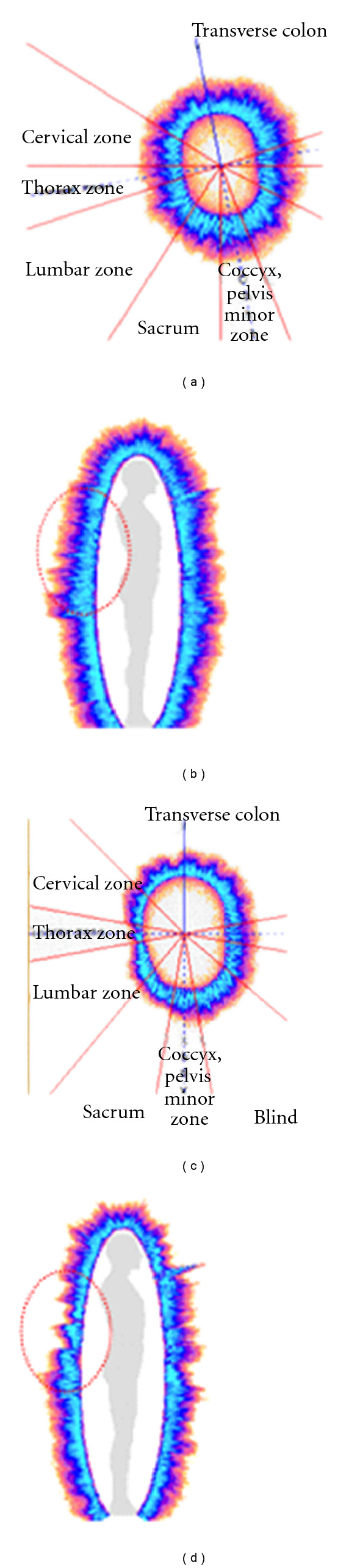
Images of the second finger of the right hand and respective lumbar and thorax zones in normal and disease states.

As a typical biometric system, the GDV biometric device is comprised of five components: a sensor, signal processing unit, data storage, a matching algorithm, and a decision process [22]. The patient places his fingertip on the sensor and the camera especially designed to capture extremely low light and takes the image of natural electrophotonic emission. The image is then processed and matched to the standard pattern. The graphic presentation that appears next on the computer screen is the result of the biometric analysis.  Figure 1 demonstrates the images of an individual who developed severe back pain over a period of time. The acupuncture points corresponding to thorax and lumbar regions have pair projection on second fingers of both hands. Image (a) shows the picture of electrophotonic emission of the second finger of right hand of a patient in a normal functional health condition. Image (b) shows the respective zones of thorax and lumber in comparison to the overall picture of energy resource of the body. Image (c) demonstrates thorax and lumbar zones of the second finger of right hand in a disease state (severe back pain reported by a patient). Image (d) compares the respective zones to the rest of the image. The integral parameters of disease state differ from normal state: area of electrophotonic emission decreased from 16953 to 13568, average intensity of the image decreased from 86.12 to 77.75, whereas entropy increased from 1.97 to 2.03. 

In one of our studies related to [23] autism from a biometrics perspective, we hypothesized that the biometric assessment based on GDV will enable us (1) to evaluate some specific features associated with autism spectrum disorder (ASD) as well as to compare autistic children to their siblings and to controls and (2) to analyze the differences in individual values of parents of autistic children versus parents of normal children. Out of total of 48 acupuncture points present on ten fingertips of both hands and associated to organs/organ systems, autistic children differed significantly from controls (*P* < .05) in 36 images (without filter) and 12 images (with filter), siblings differed significantly from controls (*P* < .05) in 12 images (without filter) and seven images (with filter), autistic children differed significantly (*P* < .05) from siblings in eight images (without filter) and one image (with filter), fathers of autistic children differed significantly (*P* < .05) from controls in 14 images (without filter) and three images (with filter) and mothers of autistic children differed significantly (*P* < .05) from controls in five images (without filter) and nine images (with filter) acupuncture points. All compared groups have shown significant difference on both psychoemotional (images without filter) and physiological (images with filter) levels. However, the differences between autistic children and controls expressed on psychoemotional level were the most significant as compared to the other groups. Therefore, the activity of the sympathetic autonomic nervous system is significantly altered in children with autism. Another indicator of autism could be the intensity of electrophotonic emission which was higher in autistic children as compared to their siblings and controls. Thus, the average intensity of emission of autistic children was 92.41 (the average of overall 10 images), siblings was 92.37 (the average overall of 10 images), and controls was 86.6 (the average overall of 10 images). The biometric method based on GDV is a promising step in autism research that may lead towards creating a disease profile and identifying unique signature/biomarker for autism. However, further work should involve more participants in order to augment our findings.

As compared to other biometric methods used in security, GDV biometric method deals with the extraction of biological patterns for health security. The issue of the extraction of the biological signature or the combination of patterns pertaining to a particular disease which would be noninvasive, early diagnosis oriented, and to certain extent preventive is of great importance nowadays. By discussing biometrics as a science and medical biometrics in particular, we would like to draw attention to this subject matter and bring a novel and current perspective into both medicine and biometric science.

## 5. Conclusions

Medical biometrics is a branch of biometrics which deals with the identification of a disease. Integration of new computerized biometric tools into medical field will advance the automated noninvasive identification of a disease as well as improve individualized medical care. GDV may be a vehicle that may satisfy the current demands of medical biometrics to identify a disease at early stages of development which will show as an increase in entropy and deviation from the normal functional state. The results from our pilot studies ([22–24]) are very encouraging to pursue the use of GDV to identify unique biological signatures for specific diseases and also characterize the functional state of the human organs and organ systems under different scenarios. The potential intervention of the computer science would bring another translational perspective into medical biometrics. One of the perspective areas is the identification of the intensity of the electrophotonic emission in each particular sector of GDV diagram, association to the already existing diagnosis, and the development of biometric disease identification by using the computer modeling tools.
